# N-Terminal Pro-B Type Natriuretic Peptide as a Marker of Bronchopulmonary Dysplasia or Death in Very Preterm Neonates: A Cohort Study

**DOI:** 10.1371/journal.pone.0140079

**Published:** 2015-10-09

**Authors:** Anna Sellmer, Vibeke Elisabeth Hjortdal, Jesper Vandborg Bjerre, Michael Rahbek Schmidt, Patrick J. McNamara, Bodil Hammer Bech, Tine Brink Henriksen

**Affiliations:** 1 Department of Pediatrics, Aarhus University Hospital, Aarhus, Denmark; 2 Perinatal Epidemiology Research Unit, Aarhus University, Aarhus, Denmark; 3 Department of Pediatrics, Herning Regional Hospital, Herning, Denmark; 4 Department of Cardiothoracic surgery, Aarhus University Hospital, Aarhus, Denmark; 5 Department of Cardiology, Aarhus University Hospital, Aarhus, Denmark; 6 Division of Neonatology, Hospital for Sick Children, Toronto, Canada; 7 Department of Public Health, Section for Epidemiology, Aarhus University, Aarhus, Denmark; Fondazione G. Monasterio, ITALY

## Abstract

**Background:**

Bronchopulmonary dysplasia (BPD) is a serious complication of preterm birth. Plasma N-terminal pro-B type natriuretic peptide (NT-proBNP) has been suggested as a marker that may predict BPD within a few days after birth.

**Objectives:**

To investigate the association between NT-proBNP day three and bronchopulmonary dysplasia (BPD) or death and further to assess the impact of patent ductus arteriosus (PDA) on this association in neonates born before 32 gestational weeks.

**Methods:**

A cohort study of 183 neonates born before 32 gestational weeks consecutively admitted to the Neonatal Intensive Care Unit, Aarhus University Hospital, Denmark. On day three plasma samples were collected and echocardiography carried out. NT-proBNP was measured by routine immunoassays. The combined outcome BPD or death was assessed at 36 weeks of postmenstrual age. Receiver operator characteristic (ROC) analysis was performed to determine the discrimination ability of NT-proBNP by the natural log continuous measure to recognize BPD or death. The association of BPD or death was assessed in relation to natural log NT-proBNP levels day three.

**Results:**

The risk of BPD or death increased 1.7-fold with one unit increase of natural log NT-proBNP day three when adjusted for gestational age at birth (OR = 1.7, 95% CI 1.3; 2.3). The association was found both in neonates with and without a PDA. Adjusting for GA, PDA diameter, LA:Ao-ratio, or early onset sepsis did not change the estimate.

**Conclusion:**

We found NT-proBNP to be associated with BPD or death in very preterm neonates. This association was not only explained by the PDA. We speculate that NT-proBNP may help the identification of neonates at risk of BPD as early as postnatal day three.

## Introduction

Bronchopulmonary dysplasia (BPD) is the most prevalent and one of the most serious complications of preterm birth with long-term consequences including pulmonary and neurodevelopmental impairment and post-neonatal mortality [1]. The pathogenesis of BPD is multifactorial and involves inflammation, lung injury, oxygen toxicity, and genetic predisposition although the relative contribution of each and their interplay have not been fully clarified [2,3]. Great interest lies in the potential to describe biomarkers that can identify neonates with the highest risk of BPD, enabling preventative strategies and trials of early treatment to modify risk.

B-type natriuretic peptide (BNP) and the inactive by-product N-terminal pro-B type natriuretic peptide (NT-proBNP) are cleaved from proBNP. The production and section is mediated by complex integration of mechanical, chemical, neuro-humeral, and immunological inputs [4,5]. BNP have diuretic, natriuretic, and vasodilatory properties and interacts with the renin-angiotensin-aldosterone system (RAAS) and the sympathetic nervous system [6–9]. NT-proBNP is more stable in plasma samples and has a longer half-life in circulation why it is the preferred biomarker compared to BNP. Plasma NT-proBNP level is associated with echocardiographic markers of a significant PDA [10,11]. However, there is little information about the pathophysiological role of NT-proBNP and BNP in neonatal disease [12–15].

Previous data suggests an association between plasma NT-proBNP, when measured at four weeks of age, and risk of BPD in preterm neonate [13]. This possible association may relate to inflammation, volume overload or high pulmonary arterial pressure. However, as the presence of a clinically significant PDA is associated with BPD [16–18] it is possible that the PDA, at least in part, may explain the association between NT-proBNP and BPD. A high volume shunt across a PDA will lead to increased pulmonary blood flow that may cause edema, increase pulmonary pressure and induce structural changes that could lead to BPD whereas the increased pulmonary venous return of blood to the heart may cause an increase in plasma NT-proBNP levels.

Accordingly, NT-proBNP may provide additional information in the evaluation of risk of morbidity in very preterm neonates in the immediate postnatal period. However, the interpretation and application need further exploration. The aim of this study was to evaluate the association between plasma NT-proBNP postnatal day three and BPD or death and further to assess if a possible association is explained by the PDA in very preterm newborns.

## Methods

### Study population

The study population has previously been described [18]. From June 1, 2010 to February 28, 2012 all newborns with a gestational age less than 32 completed weeks at birth admitted to our level three neonatal intensive care unit (NICU) were eligible for inclusion into this study. We excluded neonates with chromosomal abnormalities or congenital heart malformations other than atrium septum defects from the study.

### Data collection and definitions

All neonates were assessed by echocardiography as part of their routine care. Blood samples were collected together with routine blood samples, to avoid extra needle sticks and excessive blood sampling. Blood sampling and echocardiography was performed on day three (median day three; IQR 3–4). Samples were stored as plasma at – 80^°^C until analyses.

The following clinical information were obtained from the patients’ medical records; antenatal steroid administration, maternal preeclampsia, mode of delivery, multiple birth, weight and gestational age at birth (based on ultrasound), Apgar score at 1 and 5 minutes, surfactant administration, early onset sepsis (seven days of antibiotics initiated within the first three days after birth) [19,20], use of inotropes (within the first three days), and packed red blood cell transfusion (within the first three days), last day with oxygen supplementation, mechanical ventilation, and nasal continuous positive airway pressure (nCPAP). The primary outcome was BPD or death. BPD was defined as the need for oxygen supplementation or respiratory support at 36 weeks of postmenstrual age [17].

### NT-proBNP measurements

NT-proBNP was measured in batch by routine immune-assay analysis on the Cobas e601 platform (Roche Diagnostics, Basel, Switzerland) at the Department of Clinical Biochemistry, Aarhus University Hospital, Denmark. Electrochemiluminescent sandwich enzyme linked immunoabsorbant assays were used. The Elecsys pro-BNP II quantitative assay has a lower and upper detection limit of 5 and 35,000 ng/l, respectively (70,000 ng/l if diluted with Elecsys Diluent Universal) [21]. The assay complies with the DANAK ISO 15189 accreditation for Clinical Biochemical Laboratories.

### Echocardiography measurements

Echo was performed by two senior pediatric echocardiographers (J Bjerre and MR Schmidt) using a Philips IE33 Ultrasound machine with a 12-MHz cardiology probe (Philips Healthcare, Andover, Massachusetts, USA. A complete echocardiography study was performed to confirm normal anatomy of the heart. Using standard neonatal windows [22] a PDA was defined as present if flow could be visualized by color Doppler. PDA diameter was measured in B mode at the most narrow point. A clinically significant PDA was defined as a PDA with a diameter of 1.5 mm or more and a small PDA as a PDA with a diameter below 1.5 mm [23,24]. The ratio of the left atrium to the aorta (LA:Ao-ratio) was determined in the parasternal long axis with M-mode using leading edge to leading edge method.

### Clinical guidelines

The management of a PDA was according to a standardized departmental guideline. In accordance with these none of the neonates received Ibuprofen, Indomethacin or had surgical ligation performed before day three.

### Statistical analysis

The distribution of NT-proBNP was skewed, and therefore natural log transformation was applied [25,26]. Data were presented as geometric mean and 95% confidence intervals (CI). Perinatal characteristics were described by number (percentage) or median values (inter quartiles or range). The cohort was divided into quartiles of NT-proBNP to evaluate the frequency of BPD or death. Receiver operator characteristic (ROC) analysis was performed to determine the discrimination ability of NT-proBNP by the natural log continuous measure to recognize BPD or death. Odds ratios (OR) and 95% CI for BPD or death were assessed in relation to natural log NT-proBNP levels. Estimates were adjusted for co-variates chosen a priori; gestational age in weeks (continuous), PDA diameter (continuous), LA:Ao-ratio (continuous), early onset sepsis (dichotomized), mechanical ventilation (dichotomized), and presence of PDA (dichotomized). Robust cluster standard errors were used in order to take into account the correlation between twins. STATA special edition version 11 (College Station, Texas, USA) was used for analyses. All tests were two-sided. P-values of less than 0.05 were considered statistically significant.

### Ethics

All parents gave informed, written consent for their child to participate in the study. The study was approved by the Central Denmark Region Committee on Health Research Ethics (journal number M-20090243), the Danish Data Protection Agency, and the National Board of Health.

## Results

**Fig 1:**
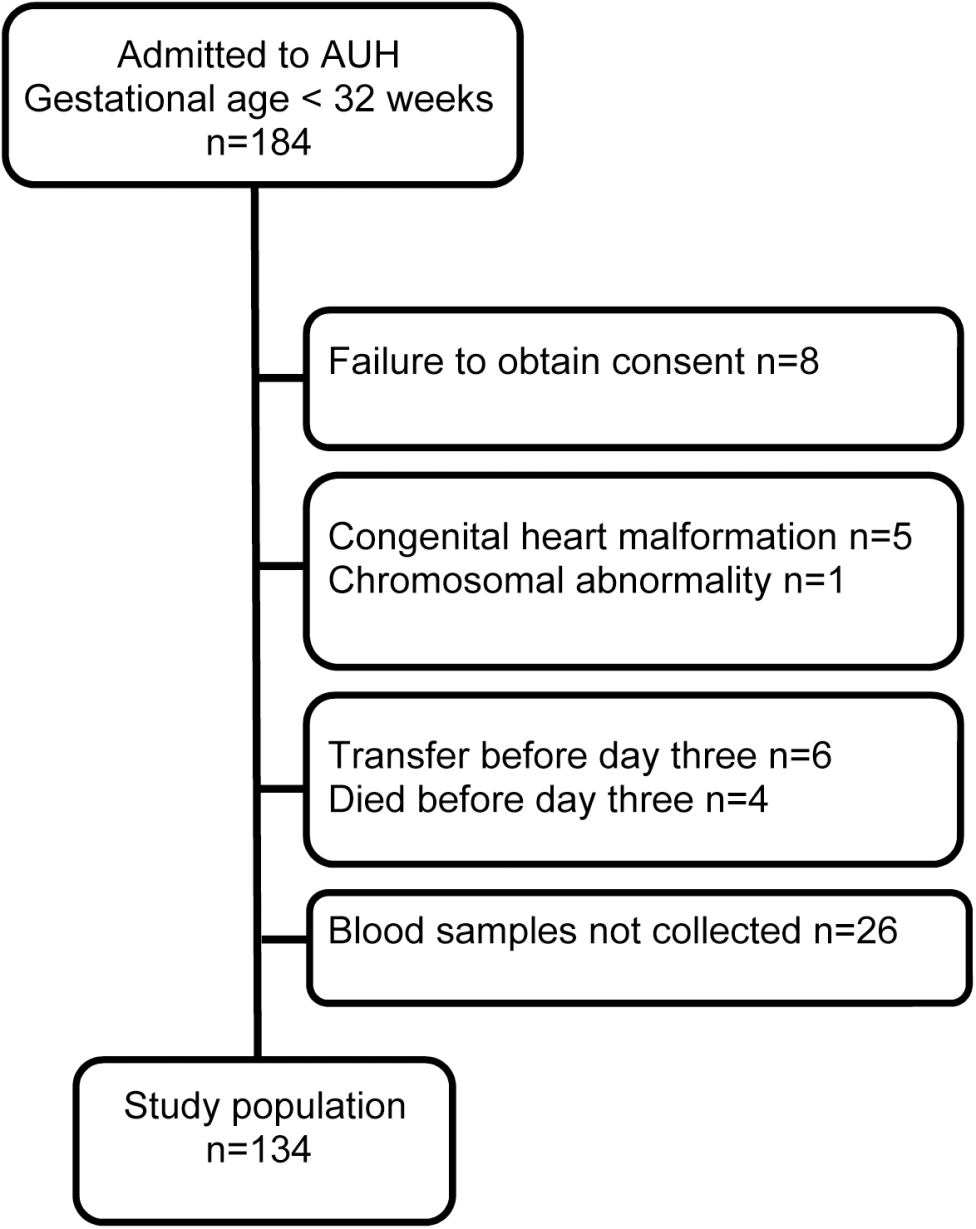
Study population based on 184 neonates consecutively admitted to Aarhus University Hospital (AUH) from June, 2010 to February 2012.

A total of 184 neonates born with a gestational age less than 32 weeks were admitted to the NICU during the study period. We consecutively obtained data on echocardiography and plasma NT-proBNP levels in 134 neonates (Fig 1). Of the 125 neonates who survived to a postmenstrual age of 36 weeks, 39 neonates (31%) were diagnosed with BPD. Neonates that were diagnosed with BPD or died had more unfavorable baseline characteristics compared to neonates with no BPD (Table 1).

**Table 1:** Characteristics of 134 neonates born before 32 gestational weeks by presence of bronchopulmonary dysplasia (BPD) or death at 36 weeks of postmenstrual age.

Characteristics	no BPD	BPD or death	p-value
	n = 86	n = 48	
GA weeks, median (range)	29 (24–31)	26 (23–31)	< 0.01
Birth weight g, median (range)	1170 (610–2090)	835 (570–1672)	< 0.01
SGA, n (%)	21 (24)	14 (29)	ns
Males, n (%)	49(57)	31(65)	ns
Singleton, n (%)	54 (63)	34 (71)	ns
Apgar at 1 min, median (IQR)	8 (5–9)	7 (5–8)	ns
Apgar at 5 min, median (IQR)	10 (9–10)	10 (8–10)	< 0.05
Surfactant, n (%)	31 (36)	30 (62)	< 0.01
Sepsis, n (%)	9 (10)	14 (29)	< 0.01
PRBC transfusion, n (%)	5 (6)	17 (35)	< 0.01
Inotropes, n (%)	5 (6)	7 (15)	ns
Mechanical ventilation, n (%)	4 (4)	16 (33)	< 0.01
PDA, n (%)	29 (34)	31 (65)	< 0.01
Clinically significant PDA, n (%)	14 (16)	20 (42)	< 0.01
Preeclampsia, n (%)	19 (22)	10 (21)	ns
Antenatal steroids, n (%)	82 (96)	33 (91)	ns
Cesarean delivery, n (%)	56 (65)	31 (65)	ns

GA gestational age, SGA small for gestational age defined as birth weight below -2SD according to the formula by Marsal er al., Sepsis defined as seven days of antibiotics initiated before day three, PRBC paced red blood cell transfusion within first three days of life, Inotropes used within the first three days of life, Mechanical ventilation day three, PDA patent ductus arteriosus day three, Clinically significant PDA if diameter ≥1.5 mm

We found that NT-proBNP levels day three were higher in neonates who developed BPD or died compared to neonates that did not (11,607 ng/l (95% CI: 8,053; 16,728) vs 3,495 ng/l (95% CI: 2,712; 4,504), p < 0.001). This finding was consistent in both neonates with a diagnosis of PDA on day three (18,901 ng/l (95% CI: 12,351; 28,924) vs 6,755 ng/l (95% CI: 4,039; 11,298), p < 0.001) and in neonates without PDA (4,770 ng/l (95% CI: 2,986; 7,621) vs 2,500 ng/l (95% CI: 1,951; 3,202), p < 0.05).

There was an increasing frequency of BPD or death from lowest to highest NT-proBNP quartiles (Table 2). Also, the frequency of PDA and especially a clinically significant PDA increased over NT-proBNP quartiles.

**Table 2:** Plasma NT-proBNP quartiles postnatal day three in relation to PDA status and morbidity in neonates born before 32 gestational weeks, Aarhus University Hospital, Denmark.

	NT-proBNP n = 134							
		Q1		Q2		Q3		Q4
Quartile range (ng/l)	500-	1,927	1,955 -	3,789	3,906-	15,977	16,414-	70,000
GA weeks, median (IQR)	30	(29–31)	28	(27–30)	28	(26–29)	27	(25–28)
PDA, n (%)	7	(21)	7	(21)	16	(48)	30	(88)
Clinically significant PDA, n (%)	0	(0)	5	(15)	6	(18)	23	(68)
Sepsis, n (%)	2	(6)	2	(6)	10	(30)	9	(26)
Mechanical ventilation, n (%)	0	(0)	3	(9)	6	(18)	11	(32)
Inotropes, n (%)	1	(3)	3	(9)	2	(6)	6	(18)
BPD or death, n (%)	3	(9)	9	(26)	13	(38)	23	(67)

NT-proBNP N-Terminal pro-Brain Natriuretic peptide maximum detection limit of 70,000 ng/l. PDA patent ductus arteriosus day three. GA gestational age. Clinically significant PDA if diameter ≥1.5 mm. Sepsis defined as seven days of antibiotics initiated before day three. Inotropes used within the first three days of life. Mechanical ventilation postnatal day three. BPD bronchopulmonary dysplasia.

**Fig 2:**
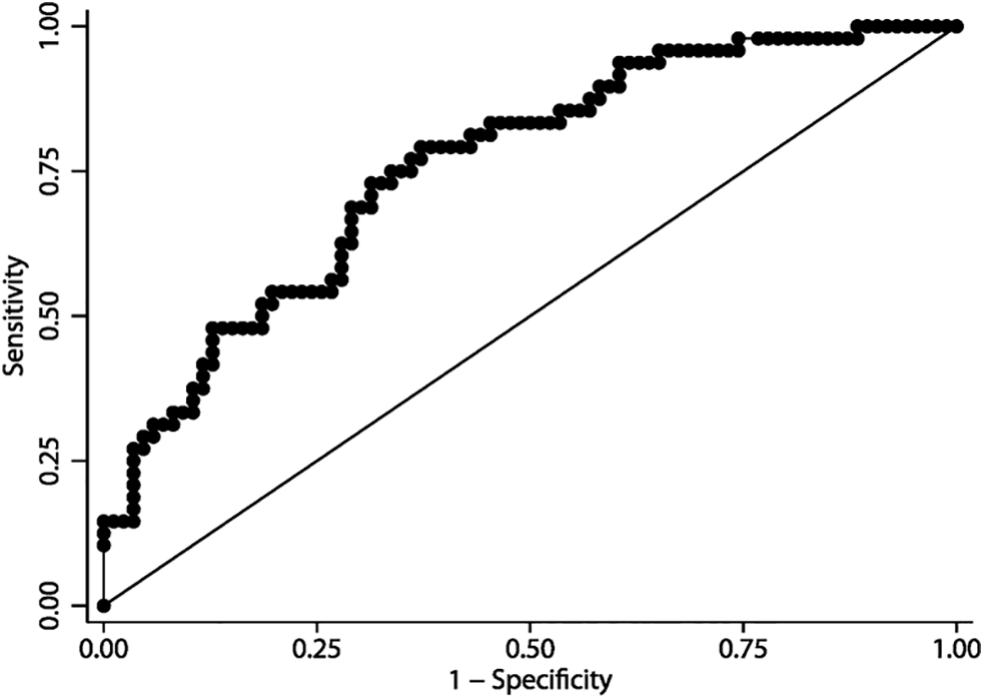
Receiver operator characteristics curve of plasma NT-proBNP day three in 134 neonates born before 32 gestational weeks to predict BPD at 36 weeks of postmenstrual age or death. Area under the curve (AUC) for NT-proBNP in predicting BPD or death (AUC = 0.76, 95% CI: 0.68; 0.84).

Fig 2 shows the receiver operating characteristic analysis distribution of plasma NT-proBNP in predicting BPD or death. The area under the curve for NT-proBNP was 0.76 (95% CI: 0.68; 0.84). The area under the curve for neonates with a clinically significant PDA was larger than the area for neonates with no PDA or a clinically insignificant PDA based on diameter (AUC day three no PDA = 0.70 (95% CI: 0.57–0.83) vs AUC clinically insignificant PDA = 0.64 (95% CI: 0.41–0.86) vs AUC clinically significant PDA = 0.82 (95% CI: 0.67–0.96).

Multivariate logistic regression analysis showed that the odds of BPD or death adjusted for gestational age increased 1.7-fold (OR = 1.7, 95% CI: 1.3; 2.3) for every one unit increase in the natural logarithm of the concentration of NT-proBNP (one unit increase in the natural logarithm is equivalent to a 2.7 multiplication of the concentration on the ordinary scale). Excluding neonates born before 26 gestational weeks (n = 23) or neonates on mechanical ventilation day three (n = 20) did not chance this estimate. In neonates with no PDA the odds of BPD or death was almost doubled for every one unit increase in the natural logarithm of the concentration of NT-proBNP when adjusted for GA (OR = 1.9; 95% CI: 1.1; 3.2) and increased by 4-fold in neonates with a clinically significant PDA (OR = 4.4; 95% CI: 1.3; 16), whereas no statistically significant association was found in neonates with a non-clinically significant PDA (OR = 1.2; 95% CI: 0.6; 2.4). Further adjusting for PDA diameter, LA:Ao-ratio, mechanical ventilation, and early onset sepsis did not influence the estimated association.

## Discussion

We found that the risk of BPD or death was associated with plasma NT-proBNP as early as postnatal day three, also after adjusting for gestational age. We found that the association between BPD or death and NT-proBNP was strongest in neonates with a clinically significant PDA on day three. Further adjusting for LA:Ao-ratio, PDA diameter and early onset sepsis did not change this association. The association was also present in neonates with no PDA, suggesting that the association between BPD or death and NT-proBNP is not only explained by the PDA.

This is, to the best of our knowledge, the first study to investigate the relation between plasma NT-proBNP postnatal day three and BPD or death. Joseph et al. reported that higher plasma NT-proBNP measured in preterm neonates at four weeks of age was associated with an increased risk of BPD [13]. This was a pilot study including only 34 neonates, born with a gestational age less than 34 weeks. Neonates requiring supplemental oxygen at four weeks postnatal age were defined as having BPD. We defined BPD in accordance with most literature as a need for supplemental oxygen or respiratory support at 36 postmenstrual weeks [12,17]. In a study including a total of 136 neonates with a birth weight below 1500 g Czernik et al. found an association between urine NT-proBNP at the age of 7 days and BPD [12]. Recently evidence, from a cohort of 60 neonates born before 32 gestational weeks, concluded that BNP was associated with BPD at the time of diagnosis [27].

Increasing evidence supports the use of NT-proBNP levels as biomarkers in screening, diagnosis, management, and follow-up of children with cardiac disease[28]. NT-proBNP levels have been found to correlated with the magnitude of a left-to-right shunt[29]. Czernik et al. also described an association between NT-proBNP and retinopathy of the preterm (ROP) [12]. El-Khuffash et al. found that high NT-proBNP and troponin T levels in neonates with a PDA were associated with an increased risk of intraventricular hemorrhage (IVH) or death [14]. They further described that combining NT-proBNP and troponin T with echocardiographic measures of a significant PDA may facilitate the identification of neonates at risk of death or poor neurodevelopmental outcome at two years of age [15].

We decided, a prior, to explore the relation between PDA, NT-proBNP and BPD further in an attempt to understand this association. NT-proBNP levels are known to increase with presence of a PDA, increased PDA diameter and LA:Ao-ratio [30,31]. Pulmonary blood flow is increased by an aortic-pulmonary shunt across a PDA, therein increasing venous return to the heart and this may increase left atrium and ventricular pressure and size [23,32]. This is known to increase synthesis and release of BNP and hence NT-proBNP [7]. Neither of the studies by Joseph et al. or Czernik et al. provided information on attributes of the PDA prior to 4 weeks of postnatal age. We found that the association between NT-proBNP and BPD or death was present regardless of the presence of a PDA on day three. In addition the estimate of the association did not change when corrected for PDA diameter or LA:Ao-ratio. However, the strongest association between NT-proBNP and BPD or death was found in neonates with a clinically significant PDA day three. This suggests that the presence of a PDA does not solely explain the increased NT-proBNP levels, but re-affirms the need to consider other aspects of hemodynamic significance as the presence of a clinically significant PDA does modify the association between NT-proBNP and BPD or death. These data align with recent reports that suggest that PDA shunt volume may be a more clinically relevant end-point of interest, rather than diameter alone which is subject to operator dependent error and does not consider transductal resistance patterns. The lack of impact of the LA:Ao-ratio on the association, may relate to the confounding effect of a large left-to-right transatrial shunt which is commonly associated with increased PDA shunt volume [33].

Presence of a PDA and especially a PDA with a diameter larger than 1.5 mm on day three is known to be associated with BPD [16,18]. Excessive pulmonary blood flow and pressure in an immature lung with ongoing maturation of alveolar and vascular structures may result in abnormal development of the pulmonary vessels [34]. However, a causal relationship between PDA and BPD has not been proven [18]. Infection [35] and pulmonary inflammation may play a crucial role in the development of BPD [36]. In vitro studies have suggested that cytokines are involved in NT-proBNP release [37,38]. In adults plasma BNP levels have been found to be associated with severe sepsis [39]. We found that adjusting for early onset sepsis did not change the described association between NT-proBNP and BPD or death.

The heart exerts an endocrine function and has a role in the regulation of both cardiovascular and renal systems [4,40]. BNP has been found to increase after birth and reach a plateau at day three to four [41]. The presence of a high volume PDA shunt or sustained elevation in pulmonary blood pressure [42] as part of physiological transition from fetal to postnatal circulation may cause this increase [41]. Water and sodium handling has been found to be different at four weeks of age in neonates that develop BPD compared to neonates without BPD [43]. In very low birth weight infants higher water intake and less weight loss during the first ten days of life have been found to be associated with an increased risk of BPD [44,45]. The release of pro-BNP and hence BNP and NT-proBNP may reflect a response to volume overload and an augmentation of the RAAS system. Further investigations into these complex integrative regulative mechanisms would be of great interest in order not only to evaluate the use of NT-proBNP as a biomarker but also to describe the pathogenesis of BPD.

### Strengths and limitations

We consecutively included neonates admitted to the NICU. Echocardiography and plasma samples were collected systematically very early in life and prior to the outcome of interest. BPD or death was used as the outcome rather than BPD because death is a competing outcome with BPD [46]. BPD was defined as need for supplemental oxygen or respiratory support by 36 weeks of postmenstrual age. Often ventilator support is used in the definition of BPD, but as none of the neonates needed mechanical ventilation at this point we reported also the need for supplemental oxygen and nCPAP.

Neonates that were diagnosed with BPD or died were born at a lower gestational age compared to neonates with no BPD. To make groups more comparable we made sub-analysis excluding neonates born before 26 gestational weeks and found that the association between BPD or death and plasma NT-proBNP was unchanged.

In 26 neonates that had an echocardiography we did not obtain plasma samples. This was in some cases due to the order for the blood samples not being communicated but may possibly also have been related to neonates that were not stable enough for blood sampling and could thus affect generalizability. In five neonates plasma NT-proBNP was above the detection limit of 70,000 ng/l they were in the analysis set to the value of 70,000 ng/l. All of these neonates died or had BPD by 36 weeks of postmenstrual age.

## Conclusion

We found increased NT-proBNP levels as early as day three of life to be associated with an increased risk of BPD or death. The strongest association was found in neonates with a clinically significant PDA; however, it was also present in neonates with no PDA. The association remained unchanged after adjusting for PDA diameter, LA:Ao-ratio and perinatal characteristics suggesting that NT-proBNP may be helpful in PDA treatment selection and as an early marker of BPD in very preterm neonates.
